# Rest and Recreation

**Published:** 1920-10-30

**Authors:** 


### Rest and Recreation.

### THE KEY TO NATIONAL HEALTH.

The discussion which took place at the Church Congress on the ethics of amusements particularly interested me (writes a correspondent) because only a couple of days previously I had been talking on the subject of recreation to a very able physician, who expressed the opinion that in a general and regularly diffused system of recreation lay the secret for restoring this country to health, both physically and economically. It is a real misfortune that Professor Keith's striking analysis of the physique of the average manhood of Great Britain, though it created something very like a "sensation" at the time, should have produced so far no definite results, either in Government or local action. There was some hope for a few weeks that particularly in the large industrial centres the local authorities had been aroused to a sense of responsibility to this matter, and would take steps to introduce practical methods of dealing with the problem, particularly in the direction of providing more open-air "lungs" in which the crowded population could breathe freely and of greatly extending the opportunities for recreation among adults as well as among children. But beyond a few spasmodic schemes started in the north there is "nothing to report."

It is a great pity, because nothing in recent years has brought home more effectively to the public mind the vital importance of healthy recreation in

**THE PUBLIC WELL-BEING**—(*continued*).

a highly industrialised country than Professor Keith's report. In fact, it is not overstating the case to say that unless such compensation is provided on a national scale Great Britain will not be able to escape from a progressive deterioration which will end ultimately in its decay as a great producing race. All the tendency of modern labour legislation, as well as of labour's demands, is to shorten the hours of work, and therefore to increase the hours of rest and freedom. This tendency offers a valuable opportunity, and at the same time places upon the State the responsibility of making it possible for the hours of freedom to be employed with benefit to the physical and mental health of the people.

As Professor Dearmer said at the Congress at Southend, in debating the question of Sunday amusements, rest from work does not mean merely animal inactivity. For the large sedentary section of humanity such rest ought to take the form of outdoor games. "Loafing," he added, "is treated as a peculiarly Sabbatical act, both in villages and towns, and with it go pitch-and-toss and worse things."

That may be true, and no doubt it is a fact that idleness, as apart from rest, breeds mischief, but I am afraid the Congress speakers did not materially help to solve the larger problem by making the old conventional complaints about the misbehaviour of people with nothing to do. Sometimes, said the Bishop of Sheffield, with considerable melancholy, there came upon him "a shivering feeling" that there was a horrible likeness between what amuses the mass of the people now and what amused them in the declining days of the Roman Empire. This because a football replay in a northern town attracted fifty thousand spectators. I do not wish to discuss the ethics of the matter, though it seems to me that the Bishop's parallel is a false one; but, on the grounds of public health, it should be the business of every intelligent member of the community to encourage by all means in his power not only the practice of active games but everything in the national life which makes for existence in the open air, whether it is watching somebody else play or taking char-a-banc rides into the Yorkshire moors.

I should like to think that at the forthcoming municipal elections every candidate would be pressed and harassed into including in his local government programme a promise to insist on the following items of municipal policy: (1) More open-air spaces for recreation within easy reach of the people; (2) a sum to be set apart for the proper provision of tennis courts, cricket grounds, hockey grounds, football grounds, gymnasia, and open-air swimming baths; (3) at least one open-air school in the country for delicate and under-developed children; (4) at least one day a week set apart for the conveyance of school children into the country during the summer months at the public expense; (5) outdoor games on turf to be made a dominating feature of the school curricula; (6) the establishment of municipal leagues of sport. As a ratepayer and taxpayer who views with considerable concern the soaring activity of both rates and taxes, I should yet be quite ready to share in the extra expenditure involved by schemes of this kind, because I believe that within a very few years ratepayer and taxpayer would receive back the interest on their money up to hundreds per cent. in the increased vitality of the nation, and therefore its increasing power of production. And we should have to spend less on our workhouses, sanatoria, lunatic asylums, and all the other insignia of disease and poverty.

